# Biodegradable Cements for Bone Regeneration

**DOI:** 10.3390/jfb14030134

**Published:** 2023-02-27

**Authors:** Dachuan Liu, Chen Cui, Weicheng Chen, Jiaxu Shi, Bin Li, Song Chen

**Affiliations:** 1 Department of Orthopaedic Surgery, Orthopaedic Institute, The First Affiliated Hospital, Suzhou Medical College, Soochow University, Suzhou 215006, China; 2 Department of Orthopaedic Surgery, The Third Affiliated Hospital of Soochow University, 185 Ju Qian Road, Changzhou 213003, China

**Keywords:** biomaterials, biodegradable bone cements, calcium phosphates, calcium sulfates, bone regeneration

## Abstract

Bone cements such as polymethyl methacrylate and calcium phosphates have been widely used for the reconstruction of bone. Despite their remarkable clinical success, the low degradation rate of these materials hampers a broader clinical use. Matching the degradation rate of the materials with neo bone formation remains a challenge for bone-repairing materials. Moreover, questions such as the mechanism of degradation and how the composition of the materials contribute to the degradation property remain unanswered. Therefore, the review provides an overview of currently used biodegradable bone cements such as calcium phosphates (CaP), calcium sulfates and organic-inorganic composites. The possible degradation mechanism and clinical performance of the biodegradable cements are summarized. This paper reviews up-to-date research and applications of biodegradable cements, hoping to provide researchers in the field with inspirations and references.

## 1. Introduction

Bone defects may be caused by severe trauma, tumors and systemic disease [1,2,3]. Every year, numerous patients in the world undergo bone reconstruction treatment because of congenital defects, tumor resection and bone defects caused by fracture [4]. Currently, autologous bone graft is the gold standard for the treatment of bone defects [5]. However, its main disadvantages include a lack of supply, pain, infection and other complications [6]. Allogeneic bone transplantation is an alternative method, but there are many postoperative complications [7,8]. Therefore, artificial bone-repairing materials are promising for the repair of bone defects. Currently, most implants are made of metallic biomaterials. The most commonly used metals for medical applications are titanium and titanium alloys as well as stainless steel and cobalt–chromium–molybdenum alloys [9]. For example, Ti6Al4V and NiTi alloys are known for their high wear resistance, ductility and high hardness. Some researchers have incorporated new elements in the alloys to address their biological toxicity and excessive modulus of elasticity [10]. However, metal implants still cannot be used for filling irregular orthopedic wounds and are hardly degradable, requiring secondary surgical removal. In addition, the composition of metal implants differs greatly from that of the human body, so there is still a necessity to develop implant materials that are more compatible with human tissue and degradable.

Bone cement, as a kind of bone-filling material, has been applied in the orthopedic field. It is of great significance for filling and repairing the irregular orthopedic trauma site and is widely used in various orthopedic and dental implant fixations [11,12]. However, the most commonly used bone cements such as polymethylmethacrylate (PMMA) are nondegradable. The poor biodegradability may cause many complications, such as intramedullary hypertension and pulmonary embolism [13,14]. Therefore, the development of bone cement with good biodegradability is essential for their biomedical applications.

Biodegradable materials are the second generation of biomaterials, which play an indispensable role in tissue repair and regeneration [15,16,17]. From 2015 to 2024, the global orthopedic biomedical materials market increased from $4.3 billion to 46.5 billion [18]. Orthopedic biodegradable materials provide support, osteoconductivity and osteoinduction at the implantation site, and some of them also assist in the completion of bone tissue repair. They are gradually degraded in the organism through dissolution, enzymatic digestion, and cellular phagocytosis at a certain degradation rate during the formation of new bone. The degradation products of the materials produce either no host response or a slight host response to the organism, and in some cases, promote the bone repair. The degradation products are continuously absorbed or excreted from the tissue as it grows in, and the repaired bone tissue completely replaces the implanted material, leaving no residual material in the body when the bone repair is completed.

**Figure 1:**
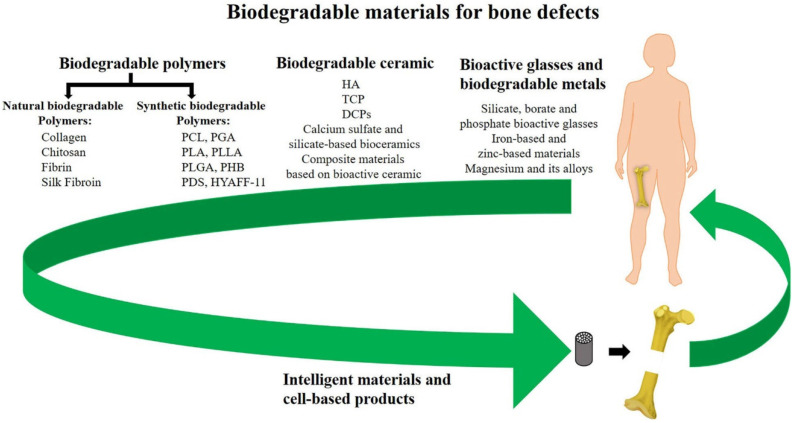
The classification of biodegradable materials used for bone defect repair [19].

According to the development status of biomaterials, biodegradable materials mainly consist of biodegradable polymers, biodegradable metal materials and biodegradable cements/ceramics (Figure 1). After implanting the materials into the defect site, the newly formed tissue grows into the implant’s interior, and some biomolecules and ions produced during the degradation can interact with the bone injury microenvironment to promote the regeneration of bone tissue. Simultaneously, the mechanical properties of the implant gradually decrease, and the biological stress of the body transfers to the new bone tissue, which prevents the stress-shielding effect and stimulates bone tissue regeneration [19,20]. However, there are some shortcomings in biodegradable bone cements, such as the mechanical strength of bone cements weakened during biodegradation, high price and inconvenient production, which all need to be continuously improved in the future [21].

A large number of examples have revealed the application prospect of degradable bone cements in the biomedical field for many years [11,19]. The subject itself has matured into an important research topic, and has achieved promising results in vitro and in vivo, which has aroused more and more interest from researchers [22,23]. In this review, we summarize the recent progress in biodegradable bone cements. Different types of biodegradable bone cements were introduced, and the mechanism of bone cements’ degradation was analyzed. Finally, the clinical application of biodegradable bone cements was summarized. Most of the previous studies summarized the mechanical properties and osteogenic ability of biological bone cement, but we focused on another important property of biological bone cement—degradability. In this review, we put forward our views on possible future directions for biodegradable bone cement research, including translating these products from laboratory to clinical. We believe that understanding the degradation process of different bone cements and optimizing the controllability of their biodegradation are the development trends of biological bone cements. This is because the biological bone cement can achieve its maximum effectiveness only when the rate of degradation matches the rate of new bone formation. It is expected that this timely review will provide a comprehensive and strong knowledge base for degradable bone cements [24].

## 2. Categories of Biodegradable Bone Cements

Bone cements are self-setting bone substitute materials, which are generally composed of powder and liquid phases [25]. In the last couple of decades, bone cements have been extensively applied in the repair and regeneration of tissue engineering due to their good biocompatibility, biodegradability and excellent osteogenesis [19,26]. To date, the most commonly used biodegradable bone cements mainly include calcium phosphate cements (CPCs), calcium sulfate cements and other composite bone cements [11]. The biggest advantage of biodegradable bone cements is that they are gradually degraded by chemical dissolution and cell absorption after implantation in the bone defect and finally replaced by the newly formed bone tissue [27].

### 2.1. Calcium Phosphate Cements

Calcium phosphate cements consist of an aqueous solution and one or several calcium phosphates [28]. Despite many formulations and compositions proposed, they can be classified into three groups according to their end products: apatite (HA, Ca_5_(PO_4_)_3_OH), brushite (DCPD, CaHPO_4_·2H_2_O) and monetite (DCPA, CaHPO_4_) [29,30]. The degradability of these three bone cements will be described below.

#### 2.1.1. Apatite Cements

**Figure 2:**
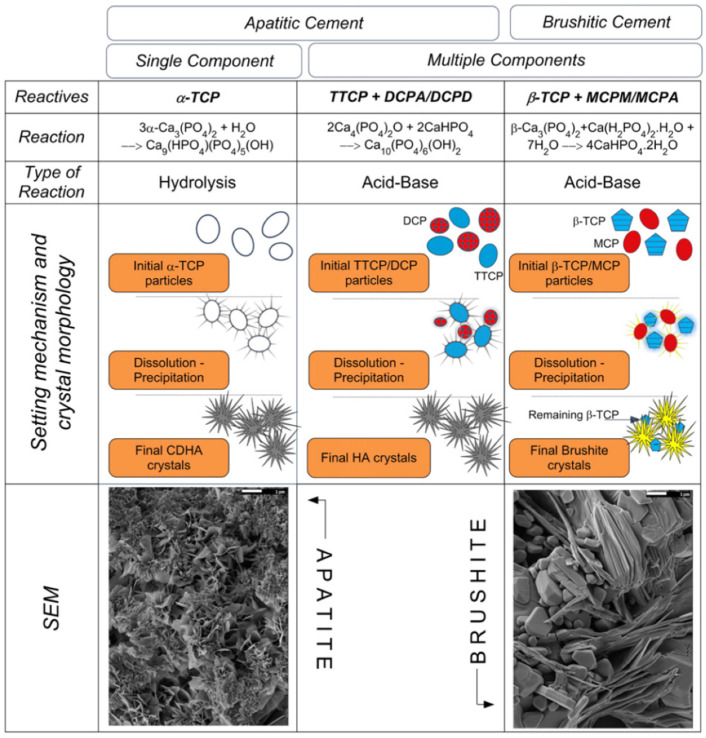
Classification of calcium phosphate cements, with examples of the most common formulations. Reprinted (adapted) with permission from [33]. 2012, Elsevier Ltd.

There are two different reaction paths for the setting of apatite cements [31]. The first is based on an acid–base reaction, where one acidic and one alkaline calcium phosphate source react to form a neutral product. Tetracalcium phosphate (TTCP) is the most frequently applied alkaline calcium phosphate source. The mixture of TTCP with an acidic calcium phosphate source (DCPA or DCPD) produces the precipitation of HA (Equation (1)) (Figure 2) [32].

2 CaHPO_4_ + 2 Ca_4_(PO_4_)_2_O → Ca_10_(PO_4_)_6_(OH)_2_ (1)

The second type of reaction is a simple hydration reaction. Calcium-deficient HA (CDHA) is prepared via the hydrolysis (Equation (2)) of a single CaP compound (Figure 2), such as α-Ca_3_(PO_4_)_2_ (α-TCP), which is applied in most clinical products.

3 α-Ca_3_(PO_4_)_2_ + H_2_O → Ca_9_(HPO_4_)(PO_4_)_5_(OH) (2)

Apatite cements are generally degraded through chemical dissolution and active degradation mediated by cellular activity, and the active degradation is dominant. Due to the limited degradation of cellular activity, HA is the least soluble and most stable of the different CPCs [34,35]. Many experiments indicated that apatite cements showed only slight degradation and new bone regeneration after being implanted in vivo for several months or even several years. The slow degradation of apatite not only hinders the regeneration of new bone, but also severely limits its clinical application.

#### 2.1.2. Brushite Cements

Brushite cements are prepared using an acid–base reaction. The components of acid–base reactions are usually composed of an acidic phosphorus source and basic calcium source [36]. Many formulations have been reported in the research. The most commonly applied formulation is monocalcium phosphate monohydrate (MCPM) (Figure 2) and β-tricalcium phosphate (β-TCP) (Equation (3)) [37,38].

Ca(H_2_PO_4_)_2_·H_2_O + β-Ca_3_(PO_4_)_2_ + 7 H_2_O → 4 CaHPO_4_·2 H_2_O (3)

Under physiological conditions, the dissolution rate of brushite cements is almost three times that of HA cements [31]. However, compared with the regeneration rate of bone, the degradation rate of brushite cements is still slightly slower. Flautre et al. studied a brushite cement applied in non-load-bearing defects [39]. After 6 months, the newly formed bone was not obviously increased and approximately half of the bone defect was still occupied by undegraded cement. Britta et al. prevented the formation of HA precipitate by adjusting the liquid–solid ratio, but the cements with two different powder-to-liquid ratios displayed only slight degradation after 10 months [20]. Maenz et al. used poly(l-lactide-co-glycolide) acid (PLGA) nanofibers to enhance the mechanical property of brushite cements. Although the addition of PLGA fiber promoted bone regeneration, the brushite cement still retained a large volume after 3 months. When brushite cements are used in bone defect repair operations, its good degradation and replacement via regenerative bone is preferred. Ideally, the biodegradation rate of brushite cements should be in line with the newly formed bone to allow the newly formed bone to gradually restore its mechanical properties.

Therefore, an urgent problem to be solved for brushite cement is to improve the degradation rate. At present, there are several methods to improve the degradation rate of brushite cements: (i) changing the powder-to-liquid ratio, (ii) improving porosity and (iii) inhibiting phase conversion [11]. These parameters could be adjusted in such a way that the biodegradation rate is line with the rate of bone formation. The most commonly used method is to introduce different pore sizes into bone cement to improve the local metabolism and the degradation rate of cement. Real et al. used an acid-based reaction between NaH_2_PO_4_ and NaHCO_3_ to fabricate CO_2_ bubbles into CPCs [40]. At 3 months, while the control CPCs still maintained their integrity, macroporous CPCs were almost completely degraded [41]. Additionally, Félix-Lanao et al. studied the effect of PLGA porogens on CPCs degradation [42]. They found that relatively low PLGA porogens were applied (e.g., 10–20 wt.%), being able still to promote CPC degradation and accelerate bone regeneration [43]. These two studies demonstrate that increasing porosity is effective to promote CPC degradation. Modulating the porosity can adjust the degradation rate of CPCs to match the rate of new bone formation, which will greatly contribute to the expansion of cements’ use. Besides improving porosity, preventing brushite cements from recrystallizing to form apatite is also an important way to increase the degradation rate [44]. Apelt et al. and Theiss et al. incorporate Mg^2+^ into the brushite cement to prevent phase conversion [30,45]. In a study, Grover et al. used brushite CPCs modified with pyrophosphate to inhibit phase conversion, causing a broader resorption and promoting new bone regeneration compared with the control group [46]. They proposed an innovative method to optimize the degradation performance of brushite cement. These two studies improved the degradation performance of brushite cement from the source of its slow degradation, enabling brushite cement to enhance its degradation performance while retaining its osteogenic ability and biocompatibility.

#### 2.1.3. Monetite Cements

Monetite is the anhydrous form of brushite. Monetite cements can be obtained by adjusting the reaction conditions of brushite cements. For example, setting brushite cements in excessively low pH conditions, in water-deficient environments, or in the presence of metallic ions, favoring monetite formation [47]. Another way of fabricating monetite cements is via the thermal dehydration of already-set brushite cements (Equation (4)):

CaHPO_4_·2 H_2_O → CaHPO_4_ + 2 H_2_O (4)

The degradation mechanism of monetite cements is the same as that of brushite, mainly divided into active absorption and via passive dissolution [48]. In a comparison between monetite cements and autologous grafts on grounds of bone healing efficiency, histomorphometry results indicated that 42% of the monetite cements were resorbed and that the newly formed bone within the implant occupied 43% of its volume [49]. This indicated that monetite cement resorption can offer a good balance between implant degradation and new bone regeneration on the premise of maintaining mechanical stability, an advantage that separated monetite from brushite and HA.

As a biodegradable bone cement with the potential to replace brushite cements, monetite cements have a very broad application prospect. Compared with brushite cements, it was found that monetite cements resorb at a faster rate than brushite in vivo. The main reason responsible for the higher resorption rate lies in that monetite is not transformed into apatite as readily as brushite [50]. Sheikh et al. designed monetite grafts of differing physical form by autoclaving and dry heating (under vacuum). Upon implantation for 3 months, both types of monetite cements showed complete resorption and no monetite converted into apatite [51]. Moreover, Tamimi et al. implanted monetite granules synthesized via autoclaving into alveolar bone defects. After six months, the amount of resorbed monetite (74%) and regenerated bone (60%) was obviously higher in comparison with the hydroxyapatite group and no apatite formation was observed in patients [52]. The monetite is an excellent calcium phosphate cement, and its advantage is that it will not recrystallize to form apatite and has a fast degradation rate. It has great research value as a substitute material for brushite.

### 2.2. Calcium Sulfate Cements

Calcium sulfate (CaSO_4_), also referred to as gypsum, is the first synthetic scaffold used for bone regeneration [53,54,55]. The application of calcium sulfate as a filler in bone defects was reported in 1892 [56]. In 1996, Wright Medical Company optimized the form and size of calcium sulfate cements and developed the bone repair product of Osteoset^®^ to promote the application of calcium sulfate in the orthopedic field. It has excellent biocompatibility and biodegradability; and the degradative products even cause no tissue inflammatory reaction [57].

The promotion of bone repair via calcium sulfate cements was first confirmed in animal experiments. Lillo and Peltier applied calcium sulfate cements to fill bone defects in a canine model and reported that calcium sulfate cements obviously promote bone regeneration covered by periosteum [58]. Radentz and Collings used calcium sulfate as plugging agents to promote bone defect repair in intrabony defects of a canine model [59]. Histological results showed that bone in the calcium sulfate-filled defects had a denser trabecular pattern. In 2001, Kelly et al. used calcium sulfate cements to repair bone defects caused by benign bone tumors, trauma and periprosthetic bone loss [60]. The percentage of bone regeneration and replacement of the calcium sulfate cements was 80% of the original defect size after 2 months. Calcium sulfate cements have also been applied in the management of acute traumatic defects. Yu et al. used injectable calcium sulfate cements to fill impacted metaphyseal defects in 31 patients with tibial plateau fractures [53]. The result showed that calcium sulfate cements improved the safety and stability of early knee motion. The advantage of calcium sulfate cement is that it is biocompatible and safe because it does not cause inflammation when implanted in the body.

However, the rapid degradation of calcium sulfate does not match the bone formation process, which limits its clinical application [61]. It is reported that calcium sulfate cement generally takes 4–6 weeks to completely degrade under the condition of good blood vessels. However, the degradation time of calcium sulfate was prolonged to 6–10 weeks in the poorly vascularized bone defect. In addition, it has been reported that osteoporosis can accelerate the degradation of calcium sulfate [62]. In normal rat caudal vertebra defects, calcium sulfate degraded about 80% in 4 weeks and almost completely dissolved in 8 weeks [63]. In the case of osteoporosis, calcium sulfate in the defects of coccygeal vertebra is completely degraded after 4 weeks. Rechenberg et al. found that calcium sulfate cements were already completely resorbed at 8 weeks in a drill hole model of sheep [64]. In order to match the rate of calcium sulfate degradation with the rate of new bone growth, many researchers combine calcium sulfate with other substances to reduce the porosity of calcium sulfate or steam treatment [65]. In short, reducing the degradation rate of calcium sulfate to be in line with the newly formed bone will be the next development direction of calcium sulfate cements.

### 2.3. Organic-Inorganic Composites

In order to further improve the properties of bone cement in various aspects, researchers often add organic components to the inorganic phase. Because of the addition of organic components, the degradation performance of composite bone cement becomes more complicated. The degradation rate of the composites mainly depends on the properties of the inorganic components in the composites [66]. However, the addition of organic components can accelerate or delay the degradation of bone cement to some extent. Therefore, adjusting the degradation rate of bone cement by adding organic components will be the future development direction for the personalized treatment of bone defects.

At present, the organic components added in composite bone cement can be divided into natural polymers and synthetic polymers [67]. Natural polymers mainly include gelatin, sodium alginate, silk fibroin and chitosan; the synthetic polymer is mainly composed of Polycaprolactone (PCL), Poly (Lactic Acid) (PLA), PLGA, and so on [68]. These polymers are often added into bone cement in different ways (polymer chain, microspheres) to regulate the degradation rate to meet the corresponding clinical needs. Habraken et al. added gelatin microspheres into CPCs to study their effect on the degradation of bone cement in vitro [69]. The results showed that the degradation curve of CPCs incorporated with gelatin microspheres decreased linearly, and the mass loss was about 5% at 3 months. Guo et al. prepared CPC/PLGA composites to explore in vivo degradation and mechanical property changes. They found that CPC/PLGA composites degraded significantly faster than the CPC group and lost 10% more weight at 12 weeks than CPC group [70].

In general, the addition of polymers enriches the types of biodegradable bone cement and improves its degradability and provides a good method for the clinical customization of personalized biodegradable bone cement.

## 3. The Mechanism of Degradability

**Figure 3:**
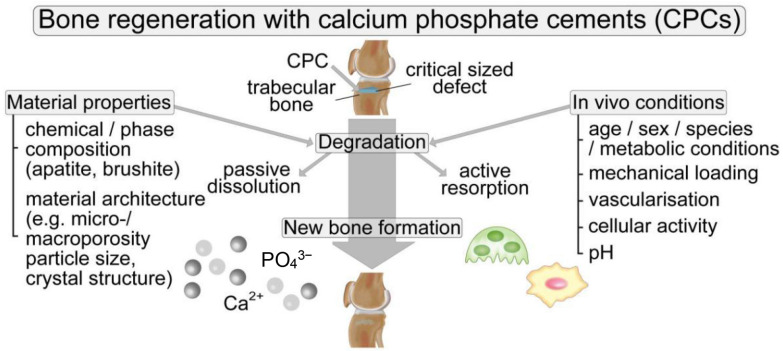
Natural process of bone repair in the fracture zone [27].

It is known that degradable bone cements can fill and repair bone defects to promote bone regeneration [71]. However, they will be gradually degraded as a foreign matter in implanted bones after implantation into the human body. The degradation modes of bone cements mainly include chemical dissolution and resorbing by the action of osteoclasts. The chemical dissolution belongs to passive degradation (Figure 3), and resorbing to active degradation [72,73].

### 3.1. Chemical Dissolution

The whole process of chemical dissolution involves physical and chemical changes without the involvement of cells [74]. The degree and speed of chemical dissolution depended on the composition, structures and the microenvironment of implant locations [37].

Among all biodegradable bone cements, the calcium sulfate cement is one of the most completely and fastest degraded cements [75]. Despite various differences between calcium sulfate hemihydrate (CSH) and calcium sulfate dihydrate (CSD), all calcium sulfates are freely soluble in physiological conditions. As the degradation of calcium sulfates, the calcium phosphate, a new phase, is formed after degradation. This phenomenon is not only the competition of two processes of precipitation and dissolution, but also a good sign for cell adhesion [57]. It is precisely because the local calcium ion concentration increases through the chemical dissolution of calcium sulfate that new bone formation is induced.

Compared with the apatite cement cements, brushite cements have been shown to degrade to a much greater extent in vivo [76]. This is mainly due to their excellent solubility at a physiological pH, which leads to degradation by chemical dissolution and active resorption by cells. The rate of chemical dissolution of CPCs is determined by the surface area, Ca/P ratio, the crystallinity and pH or perfusion with bodily liquids [11]. Dissolution occurs when brushite is placed in an environment that is under-saturated in calcium and phosphate ions, and where calcium and phosphate ions would dissolve directly. The process of dissolution stops once the solubility limit has been reached. Brushite dissolution supersaturates the environment with respect to HA, ultimately resulting in HA precipitation [37]. Because of the slow degradation rate of brushite cements, brushite cements would recrystallize to form HA during the long degradation process, which hinders its further dissolution. The resorption mechanism of monetite is the same as brushite cement implants, which is mainly mediated via chemical dissolution and cell absorption [24]. The only difference is that monetite cements dos not recrystallize to form HA, which makes monetite cements degrade faster than brushite cements.

Hydroxyapatite is insoluble in water, but can be slightly dissolved after being soaked in water for a long time [77]. Because of poorly solubility, apatite cements are primarily resorbed via active resorption through osteoclasts [61]. As for some organic–inorganic composites, they may be chemically degraded through the joint action of the active and passive methods in vivo.

### 3.2. Resorbed by the Action of Osteoclasts

Another important degradation type is active degradation mediated by cells such as osteoclasts, giant cells and macrophages [78]. The cements as implantation materials cause the inflammatory response of body’s immune system, which can induce the immune cells migration to the cements [79]. It has been reported that the degraded granules or fragments of part-degradable bone cements may stimulate the immune response, promote the secretion of inflammatory factors, such as IL-1 β, IL-6 and TNF-α, activate osteoclasts, further increase the release of inflammatory factors and activate macrophages and giant cells in bone marrow. Then the activated macrophages and giant cells will endocytose and exocytose the dissolved substances into the microenvironment between granules and cells to maintain the homeostasis of the bone environment [72]. Therefore, the cells of the mononuclear phagocytic system play a critical role in the degradation of bone implantations.

Calcium sulfate cements do not cause inflammatory reactions in vivo, so the degradable type mediated is not the main degradation type of calcium sulfate cements [57,80]. In addition, histological staining revealed that no cells are involved in implant degradation in calcium sulfate cement implants.

**Figure 4:**
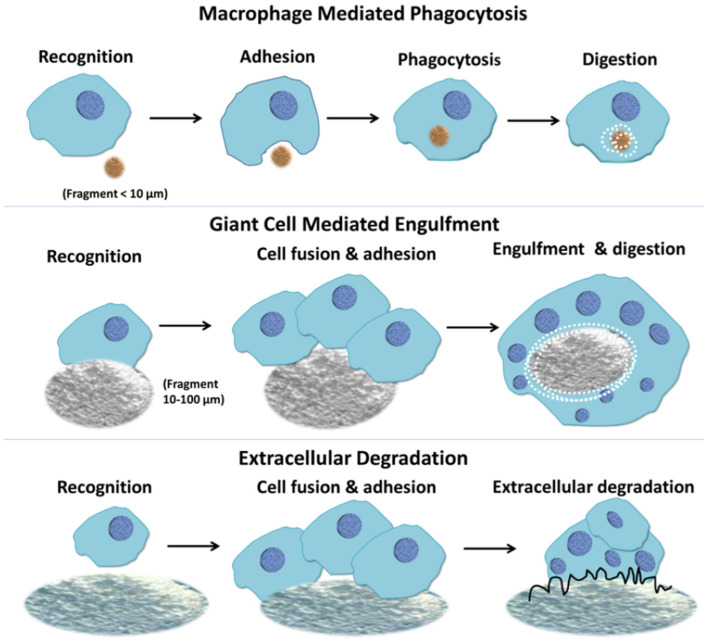
Macrophage response to biomaterials depending on the size of the implanted materials [44].

It is reported that the degradation of CPCs is mainly involved in giant cells and osteoclasts (Figure 4). But the results in vivo showed that macrophages, not osteoclasts, play an important role in the active resorption of brushite cements [45]. It has been suggested that cement disintegration and phagocytosis play a critical role, showing that brushite cement degradation may be a complicated process. If the larger cement particles were produced in the degradation process and cannot be swallowed by macrophages, the foreign matter giant cells would be responsible for degradation [81]. Sheraly et al. proved that CPC implants can be resorbed by osteoclasts, and they successfully used gastrointestinal proton pump inhibitors (PPIs) to delay osteoclast-mediated resorption of CPCs [82]. On the basis of confirming that osteoclasts participate in CPC degradation, Christian et al. quantitatively analyzed the rate of bone cement degradation involved by osteoclasts. They analyzed the active absorption efficiency of bone cements by measuring tartrate-resistant acid phosphatase (TRAP) activity and calcitonin receptor (CT-R) expression [76,83,84]. In addition, the researchers indirectly affected the degradation of calcium phosphate cement by adding different ions to the cement. Bernhardt et al. found an increased osteoclastic resorption of Cr^3+^-doped brushite cements compared to the non-incorporated brushite cement [85,86]. They also found that Co^2+^ ions promoted the formation of osteoclasts and accelerated the degradation of bone cement in vivo. Another study showed that Sr^2+^ substitution in apatite cement can effectively attenuate osteoclastic absorption and delayed the absorption of the bone cement [87]. Some studies tried to use Mg^2+^ ions and Cu^2+^ to regulate the formation or viability of osteoclasts to indirectly regulate the degradation of bone cement [84].

The active degradation of bone cements by osteoclasts plays a key role in bone cements degradation. The resorption activity of osteoclasts may be regulated by various active substances, hormones and other factors, such as interleukin, the parathyroidal hormone, calcitonin, nerve growth factor, etc. [88].

### 3.3. Recrystallized to Form Apatite

**Figure 5:**
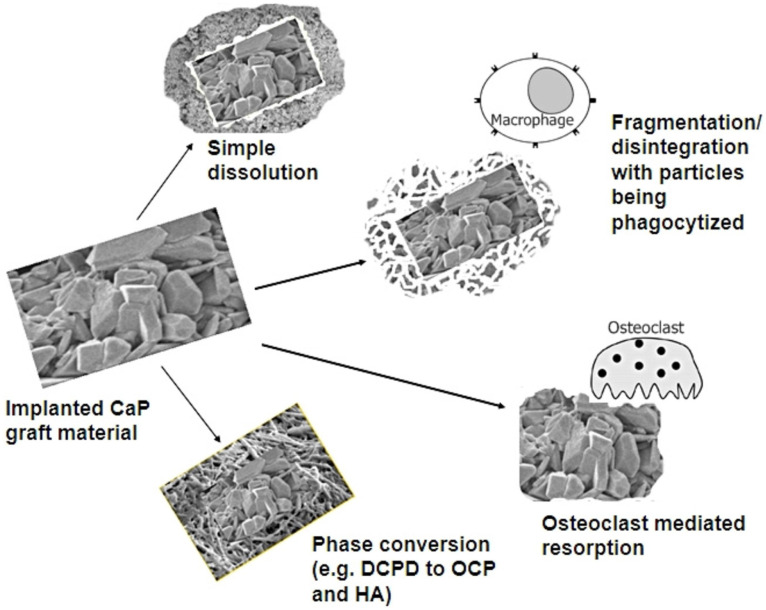
The fate of CaP biomaterials after implantation. (OCP: Octacalcium phosphate) [44].

In addition to the above two degradation modes, a small part of the calcium phosphate cement also has another degradation mode, recrystallizing, to form apatite (Figure 5). The resorption of brushite cements has been displayed to be highly unpredictable with strong dependence on many conditions [89]. One of the major conditions is phase transformation, transforming to less soluble phases such as HA, affecting the rate of resorption [50]. After the initial fast degradation of the cements, the remaining brushite is converted into insoluble apatite. It has been found that after 6 months of in vivo implantation in sheep, brushite cements completely transform into poor crystalline-carbonated apatite [90]. Many studies on the degradation of brushite cements had found that certain organic matters (i.e., citrate ions) could cause an obvious reduction in this energy barrier, and is probably involved in the process of brushite to HA conversion in vivo. In other words, physiological inhibitors of HA mineralization, such as pyrophosphate (in pyrophosphoric acid-based brushite cements), acidic amino acids and magnesium ions, can be used to prevent brushite reprecipitation as HA. For instance, Apelt et al. and Theiss et al. prevented phase transformation of brushite into insoluble phases by adding magnesium ions into the bone cement [45]. Another method to avoid the transformation into HA in vivo is to reduce the free calcium ions, which are essential for phase transformation. Schroter et al. increased the powder-to-liquid rates of cements to reduce the porosity and avoided the transformation of brushite into insoluble HA [27]. In brief, inhibiting brushite cements from transforming into insoluble the HA phase can accelerate the degradation rate of brushite cements to some extent.

## 4. Evaluation of Degradability

Ideally, CPCs are replaced with new bone formation in good time without a temporary reduction in mechanical properties [27]. However, the degradation of CPCs varies with their components and products. Therefore, different methods are required to evaluate the degradation of cement from various aspects.

In vitro, immersion and weight loss methods are commonly used to predict the degradation behavior of cements. In the measurements, the geometry and mass of the cements are detected before and after immersion [22]. Chang et al. investigated the degradation of magnesium phosphate/calcium silicate composite (MPC/CS) bone cements by submerging the samples in a Tris–HCl buffer solution. After a period of time, the weight loss of each sample was measured to assess the degradation of the bone cements in vitro [91]. The addition of CS to MPC resulted in an increased rate of degradation. The critical aspect of this method is to choose an immersion solution that better simulates the environment in vivo. For example, simulated body fluids (SBF) are widely used because this composition is similar to that of body fluids. However, minerals will deposit on the surface of the sample in SBF and thus affect the experiment results [23]. Tris–HCl, a pH 7 buffer solution, can avoid the mineralization due to its pH-balancing ability [22]. Moreover, the study of Rohanová et al. showed that unbuffered the Dulbecco’s Modified Eagle’s Medium (DMEM) could be an appropriate solution for degradation experiment [92].

Evaluation of degradability in vivo is commonly performed on tissue sections. The residual materials were observed on the stained sections, and the degradation of the materials are evaluated by comparing the residuals with the initial implanted materials. In addition, materials and surrounding tissues are assessed qualitatively and quantitatively via image analysis systems such as Micro-CT (micro-computed tomography) and SEM (scanning electron microscopy). Choll et al. established a model of osteoporosis using rodents suffering from osteoporosis [62]. Each group was packed with CaSO_4_ or CaSO_4_/CaPO_4_ cement to fill the tail defects. After 8 weeks, cement resorption profiles, bone mineral density, average cortical thickness, average trabecular thickness, average trabecular spacing and cements resorption profiles were evaluated via Micro-CT. Histologic sections were performed on spines obtained after surgery and 8 weeks. The study concluded that the pro-inflammatory and pro-osteolytic bone environments of osteoporotic disease may lead to the altered osteoconductivity and accelerated resorption of both materials.

The Norian SRS^®^, an apatite-forming commercial cement, was studied in sheep with non-loading defects in the humerus and femur [30,64]. Six months after implantation, the tissue sections showed only material degradation and marginal new bone formation. Penel et al. reported the degradation of brushite-forming cement in the femoral bone defects of sheep [90]. In the analysis of the phase of cement specimens, there was no sign of brushite detectable at 6 months or 12 months. However, a mixture of β-TCP and carbonated apatite was found, suggesting that the DCPD phase converted into slow-degrading apatite in vivo. However, the DCPD phase was transformed to slowly degraded apatite in vivo because a mixture of β-TCP and carbonated apatite was found. The phase analysis verified the unpredictability of the degradation of brushite cements.

In clinical settings, imaging observations and histopathological observations also play important roles in the study of degradability to learn the patients’ prognosis. Hillmeier et al. treated 33 patients with vertebral compression fractures via augmentation with calcium phosphate bone cement in vertebroplasty [93]. Radiomorphometric evaluation showed no significant difference in degeneration at 6 months postoperatively in comparison to patients treated with PMMA. Libicher et al. investigated the degradation of calcium phosphate bone cement in vertebral compression fractures in patients with osteoporosis after vertebral osteoplasty [94]. After 1 year, the cement implant was evaluated via CT for volume and the average degradation rate was 2 vol.%. This method exhibited high accuracy for quantifying the amount of bone cements in the vertebral body. However, given the low volume of patients in the research, the reliability of this approach necessitates more study. Heo et al. studied 14 patients with osteoporotic compressive fractures managed with vertebroplasty with brushite cements [95]. The injected CPCs exhibited unpredictable and variable morphological changes observed via radiological evaluation. Half of the implantation resulted in resorption, coagulation and fracturing. In addition, 78.6% of participants showed post-treatment advancements in vertebral compression over time. The compression has continued to progress for over 2 years. The results of this clinical study suggested that this bone cement is not mechanically strong enough to be used in vertebroplasty.

Furthermore, the degradation of CPCs cements in vivo can be evaluated by the change in mechanical properties before and after cement implantation. The degradation of cements accompanied with changes in mechanical properties [96]. The most widely studied mechanical performance of CPCs is the compressive strength [97]. Compared to cancellous bone, apatite cements exhibit similar or greater compressive strength, which is typically maintained in living organisms after implantation [27]. The new bone combined with the slow degradation of CPCs results in the formation of bone cement/bone complexes with sufficient mechanical strength and which may exceed that of intact bone trabeculae [98]. Nevertheless, the initial fast degradation of these cements may result in an unexpected reduction in mechanical properties, which may lead to failure in clinical treatment [95].

## 5. Clinical Performance of Biodegradable Cement

The CPCs were invented a century ago and have been widely researched as bone substitutes over the past 40 years [99]. A number of commercial products of calcium phosphates are available nowadays. In clinical applications, CPC is primarily used to treat patients with non-weight-bearing bone fractures to promote the recovery of joint function around the fracture. Transtemporal approaches to the petrous apex and CP (cerebellopontine) angle are standard procedures in the armamentarium of the neurotologist. Recently, calcium phosphate cement (hydroxyapatite) has been used to close cranial defects in several medical centers, as part of an FDA-IDE (Food and Drug Administration-Investigational Device Exemption) study in human subjects [100]. The results showed that apatite cement had the potential to become a standard tool for the surgical management of the skull base and temporal bone defects. Marcio et al. evaluated the efficacy and safety of HA as a highly inlaid graft on intact skulls. In 166 BFOA (bi-fronto-orbital advancement) and 19 secondary cranioplasty procedures with high inlay HA, only 1 patient had complications [101]. β-TCP was also widely used clinically as a bone repair material for maxillary sinus elevation. Suba et al. investigated the therapeutic effect of maxillary sinus elevation using β-TCP as a bone graft [102]. The results showed no statistical difference in the new bone density, microstructure of the osteo–graft material interface in the graft area between the β-TCP and autologous bone groups after 6 months. Furthermore, β-TCP degradation was significantly lower than that of the grafted autologous bone.

Apatite CPCs are used for the treatment of vertebroplasty or vertebroplasty due to their high compressive strength in CPC bone cements [27]. Nakano et al. investigated the treatment of apatite-forming CPC Biopex^®^ and Biopex-R^®^ in the clinical management of osteoporotic vertebral compression fractures after vertebroplasty [103]. After follow-up over 2 years, the clinical outcome was satisfactory, with maintained bone union and pain relief in patients. The CT images displayed that new bone was forming at the bone–cement interface and Biopex appears to be gradually degrade. Nevertheless, the degradation of cements and newly formed new bone were unquantified. In a study by Gumpert et al. [100], with the application of apatite cement KyphOS^®^ in osteoporosis, a considerable slow degradation was observed on CT scans [104]. The researchers indicated that the cement was biocompatible and osseointegrated well, as demonstrated via biopsies performed one and a half years later. In addition, the cement appeared to be gradually absorbed by the multinucleated cells, while osteoblasts formed new bone. However, the authors noted that the degradation of KyphOS was slow and may take several years. To our knowledge, there are few clinical studies on brushite cement for osteoporotic compression fractures.

Although CPCs were promising materials for bone repair, their low mechanical strength and high brittleness still limit their application in load-bearing situations [105]. Degradation that is too slow or too fast can prevent recovery, and adjusting the degradation behavior to different clinical requirements remains a major challenge [106,107]. While solving the above disadvantages, new commercial products need to be improved for more availability in the following directions: (i) improving the injectability and porosity of CPCs to optimize their osteoconductivity, (ii) using CPCs as drug carriers for the treatment of orthopedic diseases or (iii) using open macroporous CPC blocks for tissue engineering [31].

## 6. Conclusions and Outlook

Since the last century, more and more bone cements have been developed for bone regeneration. In addition to basic bone conduction and bone induction properties, these scaffolds should also be biocompatible and have a biodegradation rate similar to the rate of new bone formation to meet the time required for new bone to recover its mechanical properties. For example, researchers isolated an anhydrous form of brushite called monetite. In an experiment on the efficiency of bone healing with monetite, it was measured that 42% of the monetite cements were resorbed and that the new bone formed within the implant occupied 43% of its volume. This suggested a good balance between phosphate cement reabsorption and new bone regeneration, which helped maintain the mechanical stability of the bone defect. Biodegradable cements can be roughly divided into ceramic (calcium phosphate and calcium sulfate), polymer and composite materials. The degradation of implants in vivo depends on complex physicochemical reactions and cellular mechanisms. After the slow dissolution and particle formation of the bone cements, phagocytosis and absorption by the recruited macrophages and osteoclasts lead to the biodegradation and reabsorption of the bone cement scaffold in vivo. Although biodegradable bone cement has many clinical applications, future research is required to further understand the degradation process of different cements and optimize the controllability of biodegradation.

At present, there are still many urgent issues to be solved for biodegradable biomaterials. Basic research has been performed on the mechanical properties of biodegradable materials used for load bearing bone defects, but the control of their biodegradable properties and the mechanism of degradation are still not well understood. The ideal bone cement should provide an adhesion site for osteoprogenitor cells to deposit the bone matrix and gradually mineralize into bone. For this purpose, the cement should be slowly absorbed, allowing newly formed bone to penetrate and grow within the material. We think that the future development direction of biodegradable materials for load bearing bone defects should be the optimization of their controllability, which can be achieved by changing the porosity of material, ion doping or the preparation of composite materials. In the future, a variety of biomaterials that degrade at the same rate as new bone formation should be developed to meet the needs of patients with load-bearing bone defects.

## Data Availability

Not applicable.
